# Inflammatory Fibroblasts Promote Repair After Injury Through Epithelial Proliferation

**DOI:** 10.64898/2026.08.02.742235

**Published:** 2026-08-04

**Authors:** Luis R Rodriguez, Willy Roque Barboza, Aditi Murthy, Niran Hadad, Sarah Bui, Dakota L Jones, Yaniv Tomer, Charlotte H Cooper, Ana Reineberg, Rea Chroneos, Evan T Hoffman, Srafel Mulugeta, Jeremy Katzen, Jonathan A Kropski, Nicholas E Banovich, Michael F Beers

## Abstract

Fibroblast heterogeneity after lung injury is a well observed phenomenon made highly relevant by the widespread application of single cell RNA-sequencing. The characterization of homeostatic and injury associated states has led to the identification of a population of fibroblasts that emerge during inflammation and express cytokines that may potentially amplify the inflammatory circuit. However, whether these cells actively contribute to inflammation or serve an alternative function within the broader injury-repair cascade remains unclear. By integrating several robust murine lung injury data sets we establish the persistence of the inflammatory fibroblast across multiple injury models and identify a role for these cells in lung repair after injury through effects on alveolar epithelial proliferation. We validate this observation in-vivo using a genetic model of spontaneous lung fibrosis and in-vitro with mixed alveolar organoid cultures of various homeostatic and injury associated fibroblasts wherein we identify a mesenchymal-epithelial BMP signaling axis as a key driver of the AT2 cell injury repair response. Finally, we present supporting evidence from human disease, reinforcing the relevance of this fibroblast subset in pathological settings. These findings extend critical observations made prior to the single-cell era and contribute to our evolving understanding of fibroblast heterogeneity as a key feature of lung repair.

## Full Text Availability

The license terms selected by the author(s) for this preprint version do not permit archiving in PMC. The full text is available from the preprint server.

